# Factors associated with mortality in rheumatoid arthritis-associated interstitial lung disease: a systematic review and meta-analysis

**DOI:** 10.1186/s12931-021-01856-z

**Published:** 2021-10-11

**Authors:** Meihua Qiu, Jing Jiang, Xueyuan Nian, Yutie Wang, Pengfei Yu, Jie Song, Shenchun Zou

**Affiliations:** 1 Department of Respiratory and Critical Care Medicine, Yantai Yuhuangding Hospital, Affiliated with the Medical College of Qingdao, 20 Yuhuangding East Road, Yantai, 264200 Shandong China; 2 Department of Gastroenterology, Yantai Yuhuangding Hospital, Affiliated with the Medical College of Qingdao, 20 Yuhuangding East Road, Yantai, 264200 Shandong China; 3 Department of Rheumatology and Immunology, Yantai Yuhuangding Hospital, Affiliated with the Medical College of Qingdao, 20 Yuhuangding East Road, Yantai, 264200 Shandong China

**Keywords:** Interstitial lung disease, Rheumatoid arthritis, Mortality, Meta-analysis

## Abstract

**Background:**

Interstitial lung disease (ILD) is a common and potentially life-threatening complication for rheumatoid arthritis (RA) patients. However, there is a lack of clear prognostic factors in rheumatoid arthritis-associated interstitial lung disease (RA-ILD) patients. The purpose of this study was to complete a systematic review and meta-analysis of the factors associated with mortality in RA-ILD patients.

**Methods:**

Medline, EMBASE and the Cochrane Library were searched up to September 1, 2020. The Newcastle–Ottawa Scale (NOS) was applied to assess the methodological quality of the eligible studies. Study characteristics and magnitude of effect sizes were extracted. Then, pooled hazard ratios (HRs) with the corresponding 95% confidence intervals (CIs) and pooled risk ratios (RRs) with 95% CIs were calculated to assess the factors associated with mortality in RA-ILD.

**Results:**

Twenty-three of 3463 articles were eligible, and ten factors associated with mortality for RA-ILD were evaluated in the meta-analysis. Older age (HRs = 1.04, 95% CI 1.03–1.05), male sex (HRs = 1.44, 95% CI 1.21–1.73), having a smoking history (HRs = 1.42, 95% CI 1.03–1.96), lower diffusing capacity of the lung for carbon monoxide (DLCO)% predicted (HRs = 0.98, 95% CI 0.97–1.00), forced vital capacity (FVC)% predicted (HRs = 0.99, 95% CI 0.98–1.00), composite physiological index (CPI) (HRs = 1.04, 95% CI 1.02–1.06), usual interstitial pneumonia (UIP) pattern on HRCT (HRs = 1.88, 95% CI 1.14–3.10 and RRs = 1.90, 95% CI 1.50–2.39), emphysema presence (HRs = 2.31, 95% CI 1.58–3.39), and acute exacerbation of ILD (HRs = 2.70, 95% CI 1.67–4.36) were associated with increased mortality in RA-ILD, whereas rheumatoid factor (RF) positive status was not associated.

**Conclusions:**

Through this systematic review and meta-analysis, we found that older age, male sex, smoking history, higher CPI, lower DLCO% predicted, lower FVC% predicted, UIP pattern on HRCT, emphysema presence and acute exacerbation of ILD were associated with an increased risk of mortality in RA-ILD.

**Supplementary Information:**

The online version contains supplementary material available at 10.1186/s12931-021-01856-z.

## Background

Rheumatoid arthritis (RA) is an autoimmune, inflammatory arthritis with extra-articular manifestations in up to 50% of patients [1]. Among those manifestations, clinical and subclinical interstitial lung disease (ILD) are the most frequent extra-articular manifestations with increased morbidity and mortality, and it can be detected in 10–60% of RA patients [2, 3]. Rheumatoid arthritis-associated interstitial lung disease (RA-ILD) has a heterogeneous clinical presentation and disease course. Despite an increasing recognition of RA-ILD and growing knowledge of the related pathogenesis, ILD remains a major cause of mortality with a median survival of only 3–7 years after diagnosis [4–6]. Better means of identifying and treating RA-ILD are necessary to improve the prognosis. Multiple studies have previously investigated prognostic factors of RA-ILD. However, these findings were derived from retrospective studies with small sample sizes and the importance of their findings was unclear in isolation.

Identifying the factors consistently associated with mortality in RA-ILD would improve prognostication and provide for making better decision for clinicians in the management of these patients. Therefore, the aim of this systematic review and meta-analysis was to overcome the limitations of a primary study and identify the factors associated with all-cause mortality in RA-ILD.

## Methods

### Search strategy

We followed the Preferred Reporting Items for Systematic Reviews and Meta-Analyses (PRISMA) guidelines throughout this review [7]. We searched the Medline, EMBASE and Cochrane databases, and the Cochrane Library [the Cochrane Database of Systematic Reviews and the Cochrane Central Register of Controlled Trials (CENTRAL)] through September 1, 2020. Databases were searched and data abstracted by two authors working independently. We used subject headings and text words related to the study population to finish the search. The reference lists of eligible studies and relevant review articles were also hand-searched to find additional reports. The detailed search strategy is provided in Additional file 3: Table 1 in the online additional material.

### Study selection

Two authors independently evaluated each study for eligibility, sequentially reviewing the title, abstract and full text of each publication. Studies were included if they identified predictors of mortality in adults with RA-ILD, were published in English and enrolled at least 10 patients with RA-ILD. The primary outcome was all-cause mortality. Case reports, editorials, letters, review articles and conference proceedings were not considered. If RA-ILD patients overlapped between two studies, the study with the larger sample size was prioritized in the analysis. Any uncertainties or disagreements between two authors were resolved by discussion and consensus.

### Data extraction and quality assessment

Two authors independently extracted data from the included articles based on a predefined data extraction form. Extracted data included the first author’s name, year of publication, study location, study design, sample size, demographic features of the subjects, outcomes, potential prognostic factors and their effect estimates. The methodological quality of each cohort or case–control study was assessed using the 9-point Newcastle–Ottawa scale (NOS). Studies were of high quality if the score was ≥ 7. Two investigators assessed the quality of the studies through consultations to reach consensus.

### Statistical methods

All of the statistical analyses were performed using STATA 13.0 software (Stata Corp. LP, College Station, TX, USA). Predictors of mortality in RA-ILD in more than one of the selected studies were quantified by calculating pooled hazard ratios (HRs), pooled risk ratios (RRs), and 95% confidence intervals (CIs) using Mantel–Haenszel fixed-effects or DerSimonian–Laird random-effects models. Only unadjusted effect estimates of potential prognostic factors were combined and the effect estimates derived from multivariate models were described qualitatively. The results from the fixed effects model were presented only when there was no heterogeneity between studies; otherwise, the results from the random-effects model were presented. Heterogeneity between studies was assessed using I^2^ statistics, and statistical significance was considered with a P value of < 0.05 [8]. Forest plots were used to display the results from the individual studies and the pooled estimates. The potential for publication bias was evaluated by funnel plots and Egger’s test if 5 or more studies were available for meta-analysis [9]. If publication bias was present, the trim and fill method was used to verify the asymmetric funnel plot [10]. If combing data were deemed inappropriate, the results were reported qualitatively (due to a small number of studies or substantial clinical or methodological diversity). P-values < 0.05 were considered statistically significant.

## Results

### Search strategy

**Fig. 1:**
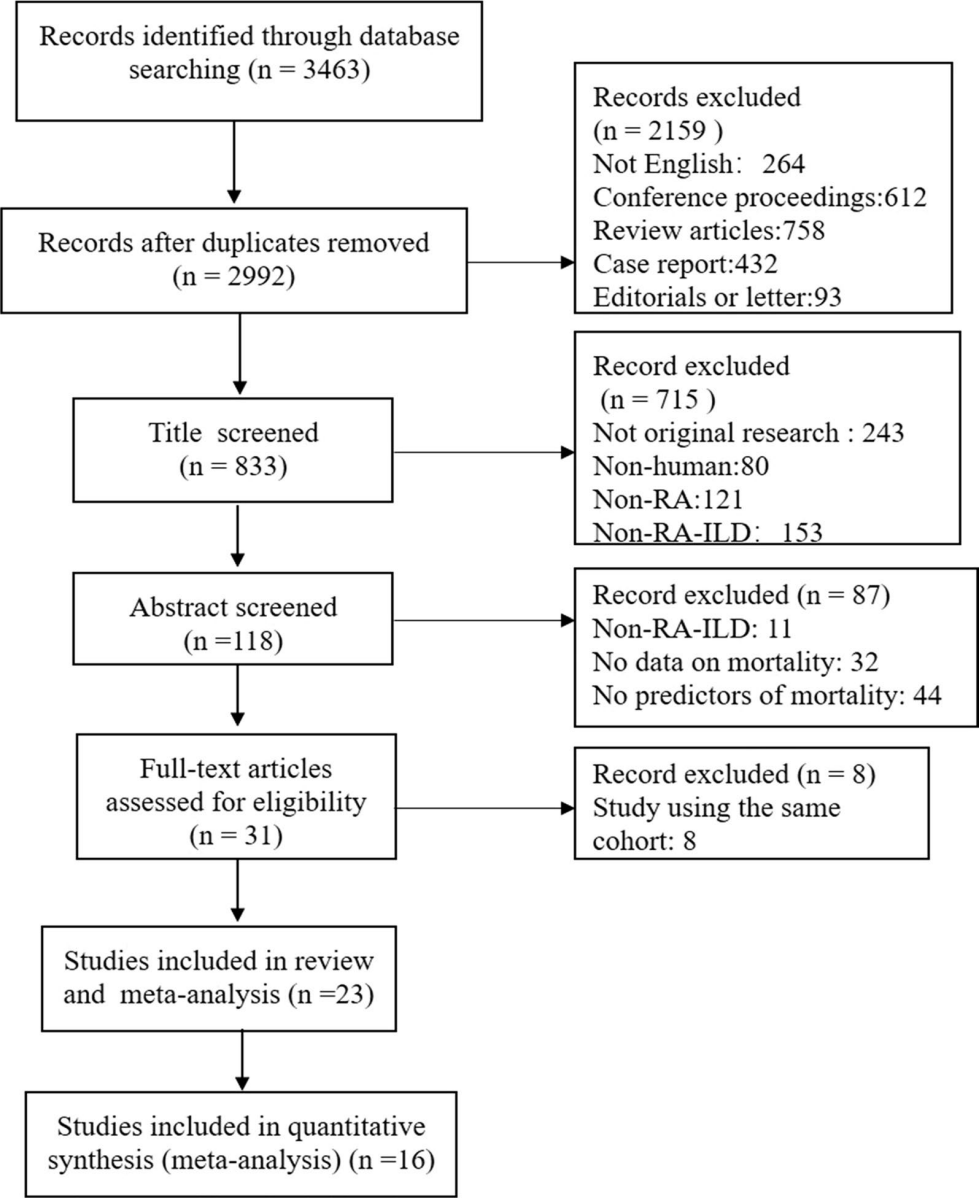
The study flow diagram

A total of 3463 reports were identified through Medline, EMBASE, and the Cochrane Library. After excluding 471 duplicates, 264 non-English records, 1895 reports of ineligible types (consisting of 612 conference proceedings, 758 review articles, 432 case reports and 93 editorials or letters) and 715 irrelevant articles, the remaining 118 reports were screened as full-texts. Of these reports, 87 were excluded due to a lack of data on RA-ILD in 11 articles, no data on mortality due to RA-ILD in 32 articles, and no data on predictors of mortality in 44 articles. Thus, eight articles using the same study cohort were excluded [11–18]. Finally, 23 studies were eligible for this systematic review and meta-analysis and 16 studies were included in the quantitative synthesis [6, 19–40] (Fig. 1).

### Study and subject characteristics

The characteristics of the included studies are shown in Table 1. The 23 studies included two prospective cohort studies [19, 22], 13 retrospective cohort studies [6, 23, 24, 27, 28, 30, 31, 33–36, 38, 40], and eight case–control studies [20, 21, 24, 26, 29, 32, 37, 39]. The studies were from nine different countries (US, UK, Japan, Republic of Korea, Mexico, Italy, Finland, Denmark and China). Of them, the majority of studies took place in the US (n = 6) and Japan (n = 6), followed by the Republic of Korea (n = 5), the UK (n = 4), China (n = 2), Italy (n = 1), Mexico (n = 1), Finland (n = 1), and Denmark (n = 1). The majority (62.5%) were single-centre studies. The 1987 American College of Rheumatology (ACR) diagnostic criteria of RA were used in 15 studies, and the 2010 ACR/EULAR diagnostic criteria of RA were used in eight studies [41, 42]. Variable criteria were used for the diagnosis of ILD, including findings on high-resolution computed tomography (HRCT) only; surgical lung biopsy only; HRCT and surgical lung biopsy; and a combination of clinical presentation, pulmonary function tests, and HRCT findings. The methodological quality of the included study is assessed by the NOS in Additional file 4: Table 2. The studies were generally of moderate-high quality. There were a total of 3062 RA-ILD patients in the included studies, with 932 deaths. The characteristics of the included study subjects are shown in Additional file 5: Table 3.

**Table 1:** Studies characteristics

Study	Country	Study design	Enrolment years	Inclusion criteria	No.RA-ILD/death	Follow-up lengths (years)
Wolfe (2007)	Multicentre (US)	Prospective cohort study	2001–2007	RA by 1987 ACR criteria; ILD diagnosed by physician and/or hospital records ICD-9 codes	100/27	Mean 3
Dixon (2010)	Multicentre (UK)	Case–control study	2001–2008	RA diagnosed by a physician; ILD diagnosed by physician and/or hospital records ICD-9 codes	367/84	Median 3.8
Kim (2010)	Multicentre (US)	Case–control study	2001–2008	RA by 1987 ACR criteria; ILD diagnosis by HRCT	82/8	Median3.9
Koduri (2010)	Multicentre (UK)	Prospective cohort study	1986–1998	RA by 1987 ACR criteria; ILD diagnosed by clinical, chest X-ray and HRCT finding	52/39	Median 10
Tsuchiya (2011)	Single-centre (Japan)	Retrospective cohort study	1996–2006	RA by 1987 ACR criteria; ILD diagnosed by HRCT finding and/or biopsy	144/71	Median 4.5
Nakamura (2012)	Single-centre (Japan)	Retrospective cohort study	1989–2009	RA by 1987 ACR criteria; Surgical lung biopsy-proven ILD	54/9	NA
Solomon (2013)	Multicentre (US)	Retrospective cohort study	1977–1999	RA by 1987 ACR criteria; Surgical lung biopsy-proven ILD	48/40	Median 3.7
Solomon (2016)	Single-centre (US)	Retrospective cohort study	1995–2013	RA diagnosed by rheumatologist ILD diagnosed by HRCT finding	137/54	Mean 4.8
Zamora-Legoff (2016)	Single-centre (US)	Retrospective cohort study	1998–2014	RA by 1987 ACR criteria ILD diagnosed by HRCT finding	181/72	Mean 3.1
Hozumi (2013)	Single-centre (Japan)	Case–control study	1995–2012	RA by 1987 ACR criteria; ILD diagnosed by clinical presentation, pulmonary function tests, HRCT finding and lung biopsy finding	51/12	Mean 8.5
Yang (2017)	Single-centre (Republic of Korea)	Case–control study	1991–2011	RA by 1987 ACR criteria; ILD diagnosed by clinical features and pulmonary function tests, radiographic finding	77/38	Mean 10
Rojas-Serrano (2017)	Single-centre (Mexico)	Retrospective cohort study	2004–2015	RA by 1987 ACR or ACR/EULAR 2010 criteria; ILD diagnosed by HRCT finding	78/17	Mean 5.8
Jacob (2018)	Multicentre (UK, Republic of Korea)	Retrospective cohort study	2007–2014 1995–2015	RA by ACR/EULAR 2010 criteria; RA-ILD diagnosed by multidisciplinary team review	245/110	Mean 6.7
Song (2013)	Multicentre (Japan)	Case–control study	1991–2008	RA by 1987 ACR criteria; UIP diagnosed by ATS/ERS consensus classification for IIP and surgical lung biopsy	84/10	Mean 2.75
Kelly (2014)	Multicentre (UK)	Case–control study	1987–2012	RA by ACR/EULAR 2010 criteria ILD diagnosed by HRCT finding	230/32	Mean 14
San-Koo (2015)	Single-centre (Republic of Korea)	Retrospective cohort study	2004–2011	RA by 1987 ACR criteria; ILD diagnosed by clinical presentation, pulmonary function testing, and radiographic finding	24/6	NA
Nurmi (2018)	Single-centre (Finland)	Retrospective cohort study	2000–2014	RA by 1987 ACR criteria; ILD diagnosed by HRCT finding	60/33	NA
Hyldgaard (2019)	Single-centre (Denmark)	Retrospective cohort study	2004–2016	RA-ILD diagnosed by physician	102/38	Mean 3.8
Ito (2019)	Single-centre (Japan)	Retrospective cohort study	2007–2016	RA by ACR/EULAR 2010 criteria; ILD diagnosed by symptoms and HRCT finding	65/16	Mean 4.7
Yamakawa (2019)	Single-centre (Japan)	Retrospective cohort study	2012–2017	RA by ACR/EULAR 2010 criteria; ILD diagnosed by a multidisciplinary team	96/25	Mean 3.7
Kim-2 (2020)	Single-centre (Republic of Korea)	Case–control study	1995–2015	RA by ACR/EULAR 2010 criteria; ILD diagnosed by HRCT finding	153/45	Mean 4.5
Li (2020)	Single-centre (China)	Retrospective cohort study	2008–2017	RA by 1987 ACR or ACR/EULAR 2010 criteria; RA-ILD diagnosed by a work group included experienced rheumatologists and radiologists	278/53	Median 4.25
Wang (2020)	Single-centre (China)	Case–control study	2016–2019	RA by ACR/EULAR 2010 criteria; ILD diagnosed by clinical presentation, pulmonary function tests, and HRCT findings	45/4	Mean 2.1

*ACR* American College of Rheumatology; *EULAR* the European League Against Rheumatism; *RA-ILD* rheumatoid arthritis-associated interstitial lung disease; *HRCT* high-resolution CT; *ICD* International Disease Classification; *US* United States; *UK* United Kingdom; *NA* not available

### Patient-specific variables

**Fig. 2:**
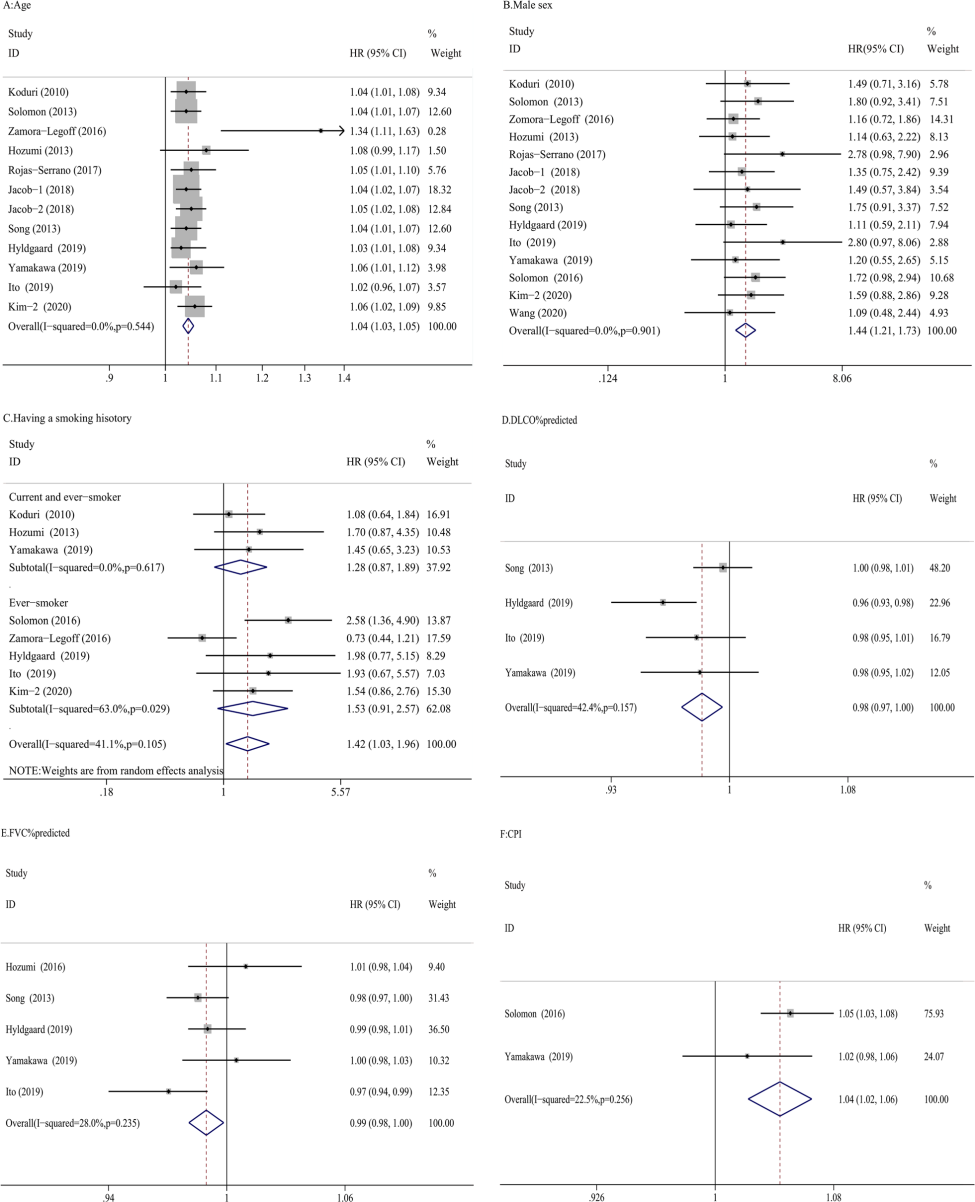
Forrest plots of the meta-analysis for age, male sex, smoking history and DLCO% predicted, FVC% predicted and CPI. Forest plots of HRs for the correlation of age with mortality in RA-ILD (**A**). Forest plot of HRs for the correlation of male sex with mortality in RA-ILD (**B**). Forest plot of HRs for the correlation of having a smoking history with mortality in RA-ILD (**C**). Forest plot of HRs for the correlation of DLCO% predicted with mortality in RA-ILD (**D**). Forest plot of HRs for the correlation of FVC% predicted with mortality in RA-ILD (**E**). Forest plot of HRs for the correlation of CPI with mortality in RA-ILD (**F**). *HRs* hazard ratios; *RA-ILD* rheumatoid arthritis-associated interstitial lung disease; *DLCO* diffusing capacity of the lung for carbon monoxide; *FVC* forced vital capacity; *CPI* composite physiological index

The patient-specific variables included age, sex, and smoking history in the meta-analysis. Fourteen studies evaluated the relationship between older age and mortality in RA-ILD. In the meta-analysis, older age was associated with increased mortality in the 11 studies reporting HRs (HRs = 1.04, 95% CI 1.03–1.04, P = 0.0001) [6, 22, 24–26, 28, 31, 35–37] (Fig. 2A). Heterogeneity across the studies was low. Visual inspection of the funnel plot showed that the funnel plot was symmetrical, suggesting that there was no publication bias (Additional file 2: Fig. 2A). In addition, the P-value of Egger’s test was 0.369 indicating that there was no evidence of publication bias. Two studies found no relationship between age and mortality in RA-ILD with odds ratio (OR) [19, 33]. However, the OR could not be pooled due to a lack of the same age threshold. Fourteen studies evaluated the impact of sex on RA-ILD mortality. There was an increased risk of mortality among men (HRs = 1.44, 95% CI 1.21–1.73, P = 0.001) [6, 22, 24–27, 30, 31, 35–37, 39] (Fig. 2B). Heterogeneity across the studies was low. The funnel plot was symmetrical and the P-value of Egger’s test was 0.093 (Additional file 2: Fig. 2B). There was no evidence of publication bias. Moreover, one study showed that female sex was associated with better survival (HR = 0.3, P = 0.008) in a multivariate model after adjusting for potential founders, including FVC% pre, DLCO% pre and UIP patterns [21]. Eight studies evaluated the relationship between a smoking history and mortality in RA-ILD. Having ever smoked and/or being a current smoker were defined as having a smoking history in this meta-analysis. Having ever smoked was evaluated in five studies, while having ever smoked and current smoking were evaluated in three studies. Having a smoking history was significantly associated with increased mortality in the meta-analysis (HRs = 1.42, 95% CI 1.03–1.96, P = 0.03) (Fig. 2C). Given the heterogeneity in defining having a smoking history, subgroup analysis was conducted. Current smoking and having ever smoked were not statistically associated with mortality in RA-ILD (HRs = 1.28, 95% CI 0.87–1.89, P = 0.205). There was also no a significant difference between having ever smoked and mortality in RA-ILD (HRs = 1.53, 95% CI 0.91–2.57, P = 0.105). Heterogeneity across the studies was low or moderate. Visual inspection of the funnel plot showed that one study fell outside the funnel plot with another borderline study, together suggesting the possibility of publication bias (Additional file 2: Fig. 2C). However, using the Egger’s test for publication bias, the P-value was 0.108 indicating that there was no publication bias.

### RA-specific variables

RA-specific variables included RF, ACPA, disease activity score (DAS28), C-reactive protein (CRP), ESR, health assessment questionnaire (HAQ) disability score and RA disease duration in the included studies. Only when these variables were collected when RA-ILD was first diagnosed, could the data be pooled. Two studies evaluated the association between positive RF status and mortality in RA-ILD [6, 27]. The included studies did not report a relevant correlation between positive RF status and mortality (HRs = 1.00, 95% CI 1.00–1.00, P = 0.009) (Additional file 1: Fig. 1). Heterogeneity across the studies was low (I^2^:0%, P = 0.659). The other three studies reported that there was no relationship between positive RF status and mortality in RA-ILD patients [22, 26, 37]. However, the data could not be pooled due to the unclear time of RF collection and different statistical methods. One study demonstrated no evidence of an association between positive ACPA status and mortality [27], whereas an ACPA titer ≥ 500 U/mL was associated with mortality (HR = 3.95, 1.12–13.93, P = 0.033) in another study [39]. However, the HRs could not be pooled due to a lack of the same ACPA threshold. One study reported that DAS28 at RA-ILD diagnosis in a multivariable model was a predictor of mortality after adjusting for potential confounders, including age, sex, calendar year of entry, disease duration, HAQ score, steroid use and methotrexate use (HR = 1.43, 95% CI 1.11–1.85) [20]. Although the DAS28 was administered at an unclear time in another study, it showed that the DAS28 was not associated with mortality in RA-ILD patients (HR = 1.01, 95% CI 1.00–1.03). Two studies collected CRP when RA-ILD was first diagnosed and explored the impact of CRP on mortality in a multivariable model [26, 30]. There was generally an increased risk of mortality for RA-ILD patients with high levels of CRP. Due to the lack of data in univariate analysis, the pooled effect could not be estimated. There was no evidence of an association between ESR and mortality in two studies (HR = 1.01, 95% CI 1–1.03, P = 0.084 and HR = 1.06, 95% CI 0.96–1.18, per 10 mm/h increase, P = 0.25) [22, 30]. Two studies showed that there could be an association between the HAQ and mortality (OR = 2.30, 95% CI 1.10–8.00, P = 0.03, and HR = 1.54, 95% CI 1.05–2.26) [19, 22]. However, it was not clear when HAQ was administered in two studies. The effect of RA disease duration at ILD diagnosis was heterogeneous across studies. Solomon et al. found that RA disease duration was not associated with mortality (HR = 1.00, 95% CI 0.97–1.02, P = 0.77) [27], whereas Zamora-Legoff et al. found that RA disease duration was associated with mortality (HR = 1.71, 95% CI 1.22–2.39, P = 0.002 per 10 years increase) [30]. Due to the difference in the estimate effect unit, the pooled effect could not be evaluated.

### ILD-physiological variables

We evaluated ILD-physiological variables including DLCO% predicted, FVC% predicted, and composite physiological index (CPI) in the meta-analysis. Only when these variables were collected when RA-ILD was first diagnosed, could the data be pooled. DLCO% predicted was associated with mortality in four studies (HRs = 0.98, 95% CI 0.97–1, P = 0.008) (Fig. 2D) [6, 26, 35, 36]. Heterogeneity across the studies was moderate (I^2^:42.4%, P = 0.157). Due to the limited number of studies, funnel plots could not be estimated. The pooled HRs (HRs = 0.99, 95% CI 0.98–1, P = 0.021) showed that FVC% predicted was significantly associated with mortality in five studies (Fig. 2E) [6, 25, 26, 35, 36]. Heterogeneity across the studies was low (I^2^:28%, P = 0.235). Visual inspection of the funnel plot was symmetrical (Additional file 2: Fig. 2D). Using the Egger’s test for publication bias, the P-value was 0.568 indicating that there was no publication bias. CPI was associated with mortality (HRs = 1.04, 95% CI 1.02–1.06, P = 0.0001) in two studies (Fig. 2F) [27, 36]. Heterogeneity across the studies was low (I^2^:22.5%, P = 0.256). Due to the limited number of studies, funnel plots could not be estimated.

### ILD-radiological variables

**Fig. 3:**
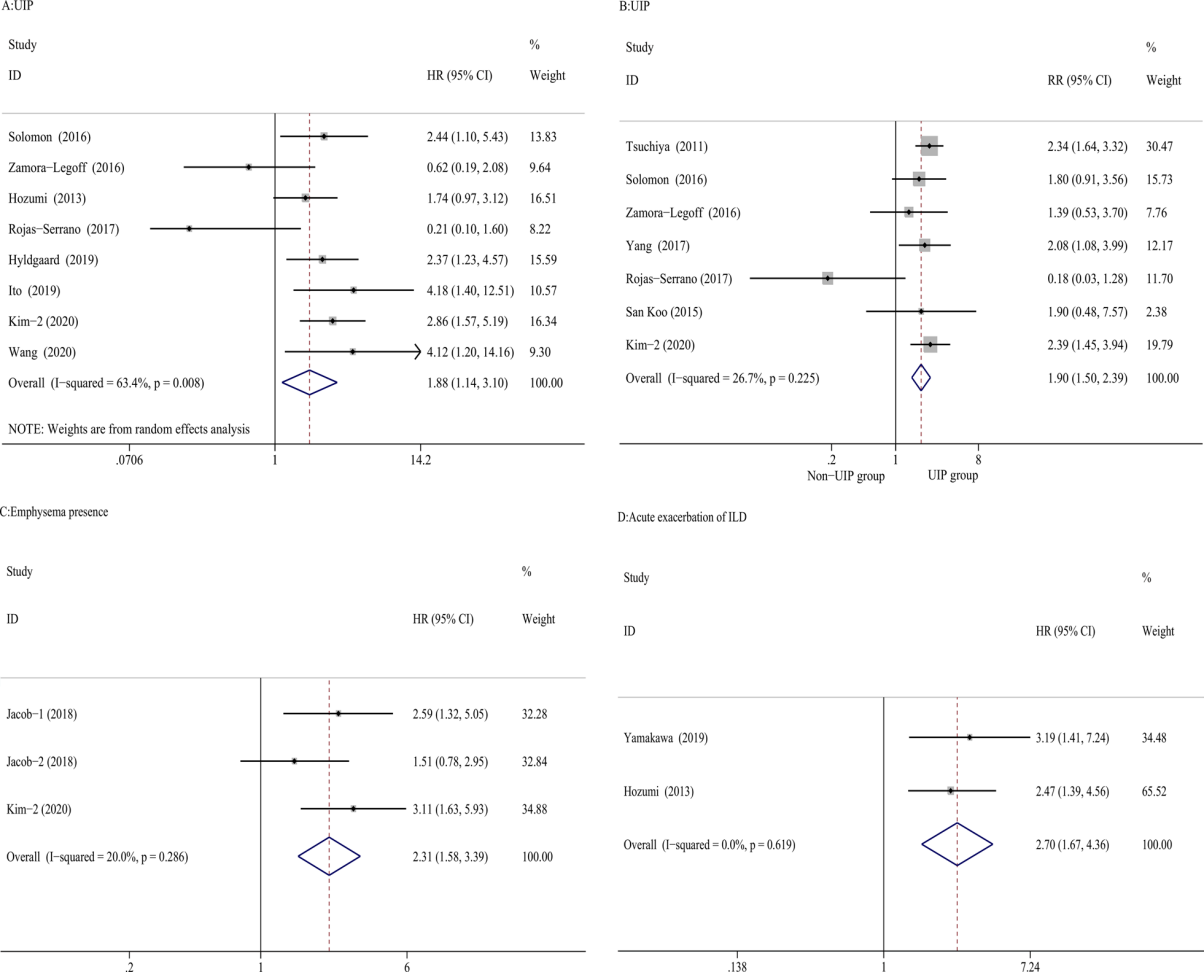
Forest plots of the meta-analysis for UIP, emphysema presence and acute exacerbation of ILD. Forest plot of HRs for the correlation of the UIP pattern with mortality in RA-ILD (**A**). Forest plot of RRs for the correlation of the UIP pattern with mortality in RA-ILD (**B**). Forest plot of HRs for the correlation of emphysema presence with mortality in RA-ILD (**C**). Forest plot of HRs for the correlation of acute exacerbation of ILD with mortality in RA-ILD (**D**). *HRs* hazard ratios; *RRs* relative ratios; *UIP* usual interstitial pneumonia; *RA-ILD* rheumatoid arthritis-associated interstitial lung disease

ILD-radiological variables, including UIP pattern, emphysema presence, and acute exacerbation of ILD, were evaluated in the meta-analysis. Due to moderate heterogeneity across the studies (I^2^:63.4%, P = 0.008), the HRs were pooled in the random-effect model (HRs = 1.88, 95% CI 1.14–3.10, P = 0.014) and the results showed that the presence of the UIP pattern was associated with mortality in the meta-analysis (Fig. 3A) [6, 25, 27, 28, 30, 35, 37, 39]. There was no publication bias according to the funnel plot and by using the Egger’s test (P = 0.327) (Additional file 2: Fig. 2E). In another seven studies, the UIP pattern was also associated with almost twofold higher mortality in the meta-analysis (RRs = 1.9, 95% CI 1.5–2.39, P = 0.0001) [27–30, 33, 37, 40] (Fig. 3B). Visual inspection of the funnel plot was asymmetric and the P-value of Egger’s test was 0.027 (Additional file 2: Fig. 2F). However, after using the trim and fill method, there was no significant difference in RRs. Although there was low publication bias, the result was stable. One study of 54 biopsy-proven rheumatoid lung disease cases also showed that the survival rates were significantly poorer in UIP patients than in non-UIP RA patients (P = 0.0452) [23]. Three studies found that emphysema presence was associated with mortality in RA-ILD patients (HRs = 2.31, 95% CI 1.58–3.39, P = 0.0001) (Fig. 3C) [31, 37]. In the included studies, the relationship between other ILD radiological variables and mortality was also evaluated, such as traction bronchiectasis, reticulation, honeycombing extent, fibrosis, ground glass score and emphysema extent. One study showed that the extent of reticulation (HR 1.144, P = 0.041) and traction bronchiectasis (HR 1.184, P = 0.030) were associated with decreased survival [34]. A large study of RA-ILD in the UK showed that extensive disease (> 20% of lung involvement) was associated with increased mortality compared with limited disease (RR = 2.17, 95% CI 1.14–4.13, P = 0.018) [32]. In another study in China, Cox hazard analysis revealed that extensive lung involvement on HRCT (> 30% of lung involved) was associated with worse survival (HR = 2.401, 95% CI 1.075–5.362, P = 0.033) [38]. However, the quantification method of chest images was not equivalent in the included studies, and the results could not be pooled. Acute exacerbation of ILD in two studies was associated with twofold higher mortality in the meta-analysis (HRs = 2.70, 95% CI 1.67–4.36, P = 0.0001) (Fig. 3D) [25, 36]. Heterogeneity across studies was low (I^2^:0%, P = 0.619). Due to the limited number of studies, funnel plots could not be estimated.

## Discussion

In this systematic review and meta-analysis, we aimed to identify factors associated with mortality among RA-ILD patients. We identified nine statistically significant factors associated with increased mortality: older age, male sex, smoking history, lower DLCO% predicted, lower FVC% predicted, higher CPI, UIP pattern on HRCT, emphysema presence and acute exacerbation of ILD. Knowledge of prognostic factors, which consist of clinical information that is easily accessible in daily clinical practice, will be of great help in developing therapeutic strategies for this intractable disease and can be very informative to patients and families in facilitating their decision-making.

Currently, the exact aetiology of RA-associated ILD is not known. However, some factors, such as older age, male sex, smoking and other serum biomarkers have been reported to be involved in the pathogenesis of RA-ILD. Among the identified prognostic factors, patient-specific factors, older age and male sex are analogous to prognostic variables in other ILD subtypes [43]. For example, older age in systemic sclerosis and male sex in idiopathic pulmonary fibrosis predict worse prognosis. The biological effect of cigarette smoking on the development of RA-ILD remains unclear. However, it is known that cigarette smoke components, such as nicotine, hydrocarbons and carbon monoxide could trigger an immune reaction that produces serum autoantibodies against multiple citrullinated proteins in the lung, in turn leading to inflammation and epithelial cell injury, ultimately resulting in ILD [44, 45].

In this systematic review and meta-analysis, we evaluated several RA-specific variables, including RF, ACPA, DAS28, CRP, ESR, HAQ disability score and RA disease duration at ILD diagnosis. If these variables were collected when RA-ILD was first diagnosed, the results could be pooled quantitatively using meta-analysis methods. As we all know, RF and ACPA can be expressed in two different ways: qualitative (positive/negative) and quantitative (titers). However, due to a lack of the same RF/ACPA threshold and different statistical methods of ACPA, the quantitative pooled results of RF/ACPA and the qualitative pooled results of ACPA could not be gotten. Although high titres of RF and ACPA are biomarkers associated with an increased risk of developing RA-ILD [46, 47], there was no relationship between RF positive status and mortality in RA-ILD patients in this meta-analysis. Moreover, ESR had no association with all-cause mortality. The ACPA, DAS28, CRP, HAQ disability score and RA disease duration were collected when RA-ILD was first diagnosed or at unclear times in these included studies. Therefore, the data could not be pooled to obtain results because of the differences in statistical time and methodology.

Pulmonary function testing (PFT) in RA-ILD patients often reveals restrictive ventilatory defects with decreases in gas exchange, and such abnormalities can be detected even in the absence of any clinical symptoms [27]. In patients with idiopathic pulmonary fibrosis, FVC and DLCO are the most sensitive parameters for assessing the clinical course. Moreover, there is a strong correlation between progressively lower values of FVC and DLCO and clinical disease severity from ILD [48, 49]. In our meta-analysis, we identified that reduced DLCO% and FVC% predicted were associated with higher mortality in RA-ILD. The CPI is a “severity variable” constructed against the morphologic severity of disease to calibrate the quantification of pulmonary fibrosis using pulmonary function tests in isolation. The study shows that the CPI correlates with the extent of pulmonary fibrosis on CT more strongly and is linked to mortality more closely than individual pulmonary function in IPF patients [50, 51]. In our meta-analysis, the CPI was associated with mortality in RA-ILD patients. Therefore, the CPI provided a valuable prognostic assessment at disease presentation.

The UIP pattern is the predominant HRCT pattern in RA-ILD and is characterized by heterogenous honeycombing at the bases and periphery of the lungs, peripheral basilar predominant reticular abnormalities and architectural distortions [21, 52]. The UIP pattern seen in RA patients is very similar to that observed in IPF patients, and it predicts worse prognosis compared with a non-UIP pattern of RA-ILD [16, 24, 53]. In never-smokers and smokers, the presence of emphysema was independently associated with the presence of honeycombing on HRCT and an HRCT UIP pattern [31]. Emphysema adversely affects prognosis by increasing the likelihood of developing pulmonary hypertension [54]. Acute exacerbation reportedly occurs not only in patients with IPF but also in patients with other interstitial lung diseases, including RA-ILD [55]. The in-hospital mortality associated with acute exacerbation in RA-ILD patients was demonstrated to be as high as that in IPF patients [25, 55]. Therefore, acute exacerbation has been suggested to be associated with mortality in RA-ILD patients as well as patients with IPF.

This systematic review and meta-analysis had several limitations. First, the studies included were heterogeneous and had variable methodological quality. Moreover, most of the studies were retrospective in design, with small sample sizes and conducted in single medical institutions. Second, the search criteria might have potentially excluded eligible studies not in English due to search being limited to the English language. Third, the diagnostic criteria of RA and the pattern of ILD in the included studies were also variable. The 1987 ACR diagnostic criteria of RA and the 2010 ACR/EULAR diagnostic criteria of RA were used in this study. The sensitivity of the 2010 ACR/EULAR criteria was greater than that of the 1987 ACR criteria, while the specificity was lower. However, the included studies were observational studies with follow-up periods, and there was little possibility of misdiagnosis. The diagnostic criteria for ILD included findings on HRCT only, surgical lung biopsy only, HRCT and surgical lung biopsy, and a combination of clinical presentation, pulmonary function tests, and HRCT findings. Currently, there are no uniform diagnostic criteria or classification guidelines for RA-ILD. Due to the risks associated with surgical lung biopsies, most patients are diagnosed and classified into the different subtypes based only on HRCT. In our study, we evaluated the correlation between the UIP pattern and mortality in RA-ILD. The UIP pattern was classified by HRCT findings in 10 of 11 included studies. UIP patterns were classified by HRCT and/or lung biopsy in one study. However, all patients with the histopathology of UIP exhibited the same HRCT pattern in this study [40]. ILD is a highly heterogenous disorder, and the diagnosis of ILD must involve the integration of multidisciplinary team meetings, including a respiratory physician, a radiologist and a rheumatologist, as well as a pathologist when applicable [56]. In addition, the difference between the factors assessed in the different studies could also limit the value of the meta-analysis. Heterogeneity across some studies was low or moderate. Sensitivity analysis was limited due to the small number of studies in this meta-analysis. Therefore, a prospective cohort study with high quality involving multiple institutions in different countries is imperative to draw a definitive conclusion.

## Conclusions

In summary, our meta-analysis identified the older age, male sex, smoking history, lower DLCO% predicted, lower FVC% predicted, UIP pattern on HRCT, emphysema presence, and acute exacerbation of ILD were associated with mortality in RA-ILD. The data should be interpreted cautiously due to the limited number of high-quality studies, substantial heterogeneity, and the limitations inherent in observational studies. This analysis could provide for better decision making by clinicians to ascertain whether early interventions targeting these factors can reduce mortality in high-risk RA-ILD patients.

## Supplementary Information

**Additional file 1: Figure 1.** Forrest plots of the meta-analysis for RF. *RF* rheumatoid factor.

**Additional file 2: Figure 2.** Funnel plots of publication bias in age (**A**), male sex (**B**), smoking history (**C**), FVC% predicted (**D**), UIP with HRs (**E**), and UIP with RRs (**F**). *HRs* hazard ratios; *RRs* relative ratios; *DLCO* diffusing capacity of the lung for carbon monoxide; *FVC* forced vital capacity; *UIP* usual interstitial pneumonia.

**Additional file 3: Table 1.** Detailed search strategy and results.

**Additional file 4: Table 2.** The quality of the included studies in the meta-analysis based on the Newcastle–Ottawa scale.

**Additional file 5: Table 3.** Patients’ characteristics.

## Data Availability

The datasets used and/or analyzed in this study are available from the corresponding author upon reasonable request.

## Abbreviations

ILD: Interstitial lung disease
RA: Rheumatoid arthritis
RA-ILD: Rheumatoid arthritis-associated interstitial lung disease
NOS: Newcastle–Ottawa Scale
HRs: Pooled hazard ratios
RRs: Pooled risk ratios
OR: Odds ratio
CI: Confidence interval
ACR/ELAR: American College of Rheumatology/European League Against Rheumatism
UIP: Usual interstitial pneumonia
FVC: Forced vital capacity
DLCO: Lower diffusing capacity of the lung for carbon monoxide
CPI: Composite physiological index
RF: Rheumatoid factor
ACPA: Anti-citrullinated peptide antibodies
ESR: Erythrocyte sedimentation rate
GGO: Ground glass opacity
HRCT: High resolution computed tomography
DAS: Disease activity score
CRP: C-reactive protein
HAQ: Health assessment questionnaire
