# Supplementary figures and images for: Factors associated with mortality in rheumatoid arthritis-associated interstitial lung disease: a systematic review and meta-analysis
Source: Respir Res. 2021 Oct 11;22:264. doi: 10.1186/s12931-021-01856-z (PMC8504109; doi:10.1186/s12931-021-01856-z)

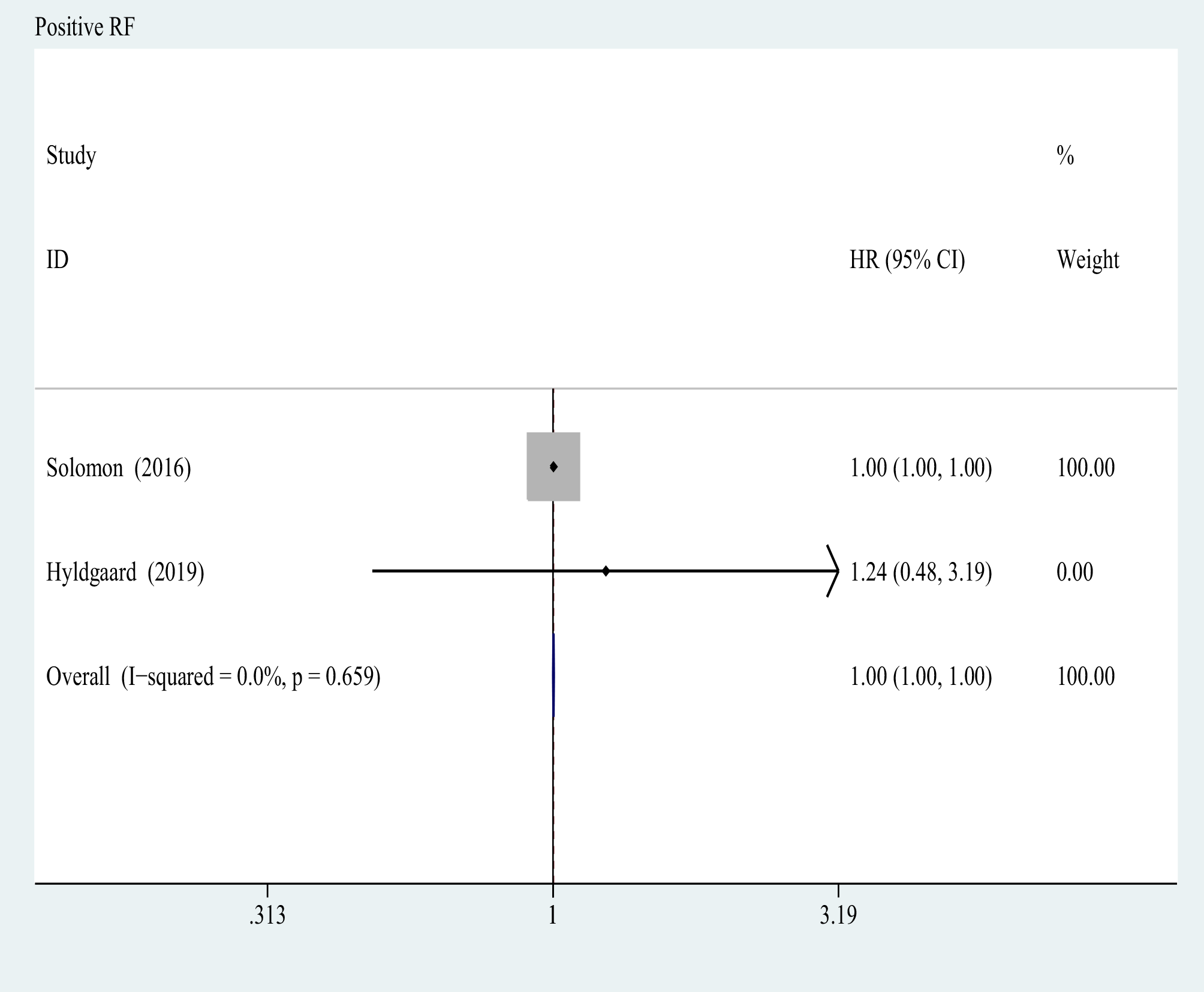

Supplement: Supplementary file 1 — Additional file 1: Figure 1. Forrest plots of the meta-analysis for RF. RF rheumatoid factor. [file 12931_2021_1856_MOESM1_ESM.tif]

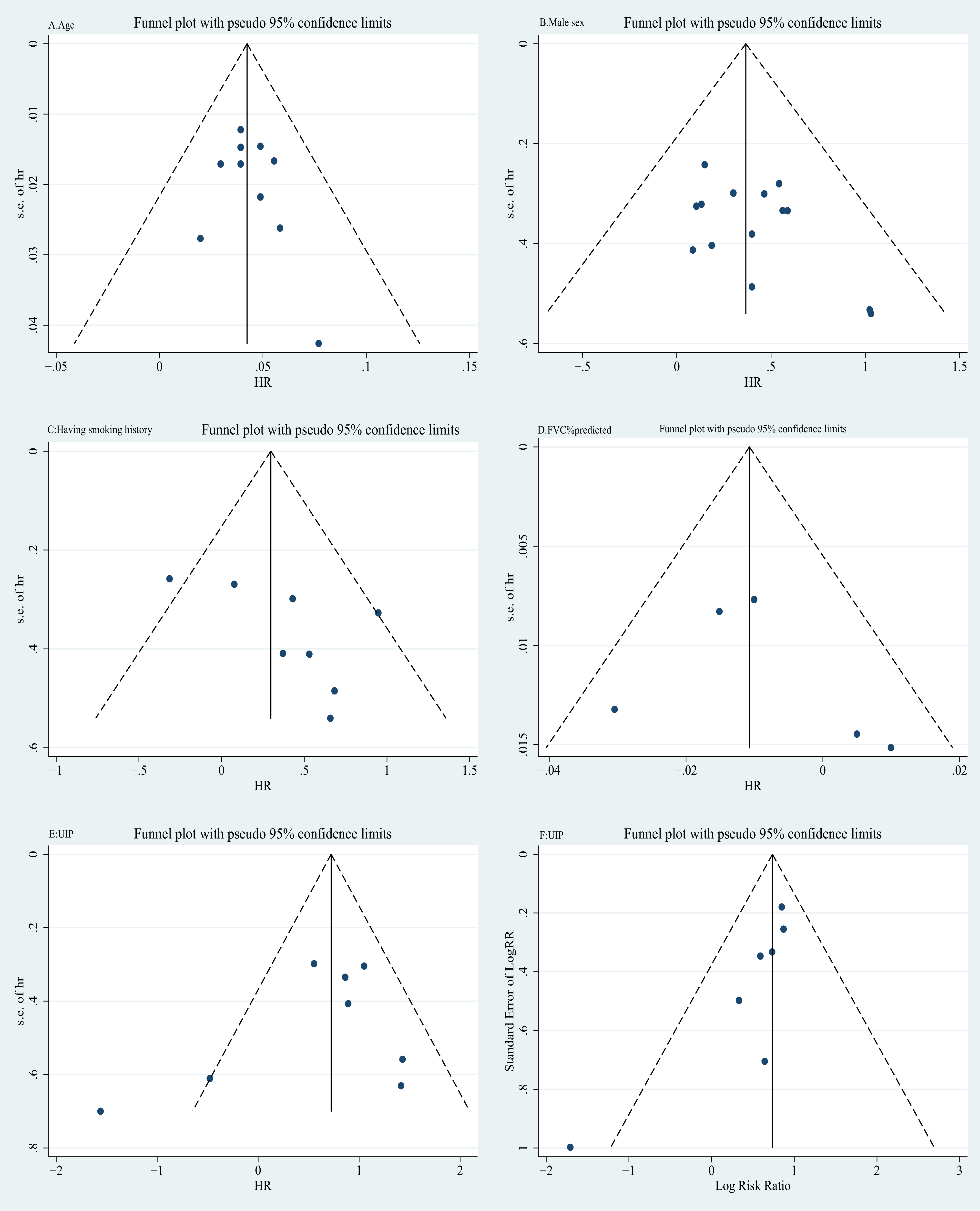

Supplement: Supplementary file 2 — Additional file 2: Figure 2. Funnel plots of publication bias in age (A), male sex (B), smoking history (C), FVC% predicted (D), UIP with HRs (E), and UIP with RRs (F). HRs hazard ratios; RRs relative ratios; DLCO diffusing capacity of the lung for carbon monoxide; FVC forced vital capacity; UIP usual interstitial pneumonia. [file 12931_2021_1856_MOESM2_ESM.tif]
