# Supplementary material for: Factors associated with mortality in rheumatoid arthritis-associated interstitial lung disease: a systematic review and meta-analysis
Source: Respir Res. 2021 Oct 11;22:264. doi: 10.1186/s12931-021-01856-z (PMC8504109; doi:10.1186/s12931-021-01856-z)
Supplement: Supplementary file 3 — Additional file 3: Table 1. Detailed search strategy and results. [file 12931_2021_1856_MOESM3_ESM.docx]

**Additional Table 1.** Detailed search strategy and results

|  | **Search term** | **Medline** |
| --- | --- | --- |
| 1 | Exp Arthritis, Rheumatoid/ | 113198 |
| 2 | rheumatoid.mp. | 144748 |
| 3 | or/1-2 | 156934 |
| 4 | Exp Lung diseases, Interstitial / | 55934 |
| 5 | interstitial lung disease.mp. | 68632 |
| 6 | Exp Pulmonary Fibrosis/ | 23637 |
| 7 | pulmonary fibrosis.mp. | 49171 |
| 8 | lung fibrosis.mp. | 63056 |
| 9 | fibrosing alveolitis.mp. | 49340 |
| 10 | interstitial pneumon*.mp. | 14797 |
| 11 | UIP.mp. | 1293 |
| 12 | usual interstitial pneumoni*.mp. | 1562 |
| 13 | NSIP | 604 |
| 14 | Non specific interstitial pneumoni*.mp. | 755 |
| 15 | or/4-14 | 123591 |
| 16 | prognos*.mp. | 880434 |
| 17 | mortality.mp. | 1279576 |
| 18 | outcome.mp. | 2521108 |
| 19 | death.mp. | 982616 |
| 20 | or/16-19 | 4362175 |
| 21 | 3 and 15 and 20 | **904** |

|  | **Search term** | **Embase** |
| --- | --- | --- |
| 1 | rheumatoid Arthritis/exp/ | 216716 |
| 2 | rheumatoid | 248943 |
| 3 | or/1-2 | 251791 |
| 4 | Exp, Interstitial lung disease / | 84050 |
| 5 | interstitial lung disease | 27488 |
| 6 | pulmonary fibrosis | 71287 |
| 7 | Exp, lung fibrosis/ | 81212 |
| 8 | lung fibrosis | 104472 |
| 9 | fibrosing alveolitis | 25428 |
| 10 | interstitial pneumon* | 30838 |
| 11 | UIP | 3107 |
| 12 | usual interstitial pneumonia | 2780 |
| 13 | NSIP | 1639 |
| 14 | Non specific interstitial pneumonia | 1589 |
| 15 | or/4-14 | 196627 |
| 16 | prognos* | 1170609 |
| 17 | mortality | 1525046 |
| 18 | outcome | 2592960 |
| 19 | death | 1288719 |
| 20 | or/16-19 | 5260012 |
| 21 | 3 and 15 and 20 | **2535** |

|  | **Search term** | **Cochrane** |
| --- | --- | --- |
| 1 | Rheumatoid arthritis/exp/, | 636 |
| 2 | Rheumatoid | 658 |
| 3 | 1 or 2 | 658 |
| 2 | interstitial lung disease | 60 |
| 3 | pulmonary Fibrosis | 87 |
| 4 | lung fibrosis | 19 |
| 5 | fibrosing alveolitis | 10 |
| 6 | interstitial pneumon* | 31 |
| 7 | UIP | 15 |
| 8 | usual interstitial pneumoni* | 7 |
| 9 | NSIP | 1 |
| 10 | Non specific interstitial pneumoni* | 3 |
| 11 | or/2-10 | 160 |
| 12 | prognos* | 3001 |
| 13 | mortality | 6058 |
| 14 | outcome | 10153 |
| 15 | death | 5624 |
| 16 | or/11-15 | 10521 |
| 17 | 1 and 2 and 16 | **24** |
