# Supplementary material for: Factors associated with mortality in rheumatoid arthritis-associated interstitial lung disease: a systematic review and meta-analysis
Source: Respir Res. 2021 Oct 11;22:264. doi: 10.1186/s12931-021-01856-z (PMC8504109; doi:10.1186/s12931-021-01856-z)
Supplement: Supplementary file 4 — Additional file 4: Table 2. The quality of the included studies in the meta-analysis based on the Newcastle–Ottawa scale. [file 12931_2021_1856_MOESM4_ESM.docx]

**Additional Table 2**. The quality of included studies in meta-analysis based on the Newcastle-Ottawa scale

3.1 Study quality of case-control studies

| **Author** | **Is the case definition adequate?** | **Representativeness of the Cases** | **Selection of Controls** | **Definition of Controls** | **Comparability of Cases and Controls on the Basis of the Design or Analysis** | **Ascertainment of exposure** | **Same method of ascertainment for cases and controls** | **Non-Response rate** | **Total scores** |
| --- | --- | --- | --- | --- | --- | --- | --- | --- | --- |
| Hozumi 2013 | ★ | ★ | ★ | ★ | ★☆ | ★ | ★ | ☆ | 7 |
| Yang 2017 | ★ | ★ | ★ | ★ | ★☆ | ★ | ☆ | ★ | 7 |
| Song 2013 | ★ | ★ | ★ | ★ | ★★ | ★ | ☆ | ☆ | 7 |
| Kim 2020-2 | ★ | ★ | ★ | ★ | ★★ | ★ | ★ | ☆ | 8 |
| Wang 2020 | ★ | ★ | ★ | ★ | ★☆ | ★ | ★ | ☆ | 7 |

3.2 Study quality of cohort studies

| **Author** | **Representativeness of the exposed cohort** | **Selection of the non exposed cohort** | **Ascertainment of exposure** | **Demonstration that outcome of interest was not present at start of study** | **Comparability of cohorts on the basis of the design or analysis** | **Assessment of outcome** | **Was follow-up long enough for outcomes to occur** | **Adequacy of follow up of cohorts** | **Total scores** |
| --- | --- | --- | --- | --- | --- | --- | --- | --- | --- |
| Koduri 2010 | ★ | ★ | ★ | ★ | ★ | ★ | ★ | ★ | 8 |
| Tsuchiya 2011 | ★ | ★ | ★ | ★ | ★ | ★ | ★ | ★ | 8 |
| Solomon 2013 | ★ | ★ | ★ | ★ | ★ | ★ | ☆ | ★ | 7 |
| Solomon 2016 | ★ | ★ | ★ | ☆ | ★ | ★ | ★ | ★ | 7 |
| Zamora-Legoff 2016 | ★ | ★ | ★ | ☆ | ★★ | ★ | ★ | ★ | 8 |
| Rojas-Serrano2017 | ★ | ★ | ★ | ☆ | ★ | ★ | ☆ | ★ | 6 |
| Jacob 2018 | ★ | ★ | ★ | ★ | ★ | ★ | ★ | ★ | 8 |
| San Koo 2015 | ★ | ★ | ★ | ☆ | ★ | ★ | ☆ | ★ | 6 |
| Hyldgaard 2019 | ★ | ★ | ★ | ☆ | ★ | ★ | ★ | ☆ | 6 |
| Ito 2019 | ★ | ★ | ★ | ★ | ★ | ★ | ★ | ☆ | 7 |
| Yamakawa 2019 | ★ | ★ | ★ | ☆ | ★ | ★ | ★ | ☆ | 6 |
