# Supplementary material for: Factors associated with mortality in rheumatoid arthritis-associated interstitial lung disease: a systematic review and meta-analysis
Source: Respir Res. 2021 Oct 11;22:264. doi: 10.1186/s12931-021-01856-z (PMC8504109; doi:10.1186/s12931-021-01856-z)
Supplement: Supplementary file 5 — Additional file 5: Table 3. Patients’ characteristics. [file 12931_2021_1856_MOESM5_ESM.docx]

Additional Table 3. Patients’ characteristics in the included studies

| Study | Patients (n)(M%) | Age(years) | Ever or current smoker, n (%) | FVC(%predicted) | DLCO(%predicted) | No. UIP /  UIP death |
| --- | --- | --- | --- | --- | --- | --- |
| Wolfe 2007 | 100（20） | 66.4 | NA | NA | NA | NA |
| Dixon 2010 | 68(40)  299(43) | 68(9)  63(10) | NA | NA | NA | NA |
| Kim 2010 | 82(51.2) | 69(10) | 59（72） | 69 | 48.5 | 20 |
| Koduri 2010 | 52（42.3） | 65(58-71) | 25（48.7） | NA | NA | NA |
| Tsuchiya 2011 | 144（41.7） | 65.2(9.8) | 54（37.5） | 70.4 | 78.9 | 57/45 |
| Nakamura 2012 | 54(55.6) | 60.3 | 29(54) | 81.7±19.7 | 88.4±19.2 | 15 |
| Solomon 2013 | 48(56.3) | 60(11.1) | 24(50) | 61±17.9 | 48±12.6 | NA |
| Solomon 2016 | 137(50) | 64.7±10.6 | 87(64) | 69.3±19.2 | 48.9±17.8 | 108/47 |
| Zamora-Legoff 2016 | 181(63) | 67.4±9.9 | 114(63) | 72.3±20.3 | 55.7±19.8 | 98/41 |
| Hozumi 2013 | 51(57) | 62 | 31(60.8) | 91.1(50.6-130) | NA | 14 |
| Yang 2017 | 77(24.7) | 59±13.3 | 68(88) | 81.2±19.7 | 68±20.3 | 32/19 |
| RojasSerrano2017 | 78(18) | 57.7± 12.2 | NA | 73.32±25.2 | 58.6±29.7 | 20/1 |
| Jacob 2018 | 245(44.9) | Never-smoker:60  Smoker:65 | 129(52.7) | Never-smoker:74·1 ± 18·5  Smoker: 76± 21.5 | Never-smoker :53.8±21  Smoker: 49.5±19·5 | 74 |
| Song 2013 | 84(52.4) | 62.6±10 | 40(47.6) | 75.1±20.7 | 66±21.8 | 84 |
| Kelly 2014 | 230(48) | 64 | 154(67) | NA | NA | 103 |
| San Koo 2015 | 24(12.5) | Nonsurvior:71(65-75)  Survivor:60(34-81) | 6(25) | Nonsurvivor:78(68-100)  Survivor: 77(40-104) | Nonsurvivor:55(32-67)  Survivor:65(34-105) | 5/2 |
| Nurmi 2018 | 60(56.7) | 66.5±11.2 | 36(59.3) | NA | NA | 36 |
| Hyldgaard 2019 | 102(46) | 68.2±10.7 | 76(74.5) | 89.6±23.3 | 54.8±16.2 | 55 |
| Ito 2019 | 65(52.3) | 72(67-78) | 35 (53.8) | 90.6(73.6-101.1) | 61.8(50.5-77.4) | 30 |
| Yamakawa 2020 | 96 (39) | 69.0±10.3 | 45(47) | 86.5±21.4 | 67.7±17.1 | 20 |
| Kim-2 2020 | 153(42.5) | 61.0 ± 10.2 | 66(43.1) | 75.6 ± 18.6 | 61.1 ± 19.5 | 59/27 |
| Li 2020 | 278(38.85) | 57.41±13.84 | 106(38.13) | NA | NA | 86 |
| Wang 2020 | 45(40) | 56.1±7.8 | 9(20) | 79.9±20.1 | 58.4±13.4 | 18 |

FVC: forced vital capacity; DLCO: diffusing capacity of the lung for carbon monoxide; UIP: usual interstitial pneumonia. NA: not available.
